# Exploratory multi-biofluid proteomic profiling of acute exercise responses in plasma, tear fluid, and nasal secretions

**DOI:** 10.3389/fphys.2026.1864411

**Published:** 2026-09-23

**Authors:** Mingjie Sun, Jing Cao, Maodi Liang, Tiantian Liang, Huilin Yao, Qintong Fei, Qinghua Cui

**Affiliations:** 1 School of Sports Medicine, Hubei Key Laboratory of Exercise Training and Monitoring, Wuhan Sports University, Wuhan, Hubei, China; 2 Department of Biomedical Informatics, State Key Laboratory of Vascular Homeostasis and Remodeling, School of Basic Medical Sciences, Peking University, Beijing, China

**Keywords:** data-independent acquisition, exercise proteomics, multi-biofluid analysis, nasal secretions, plasma, tear fluid

## Abstract

**Background:**

Exercise induces broad physiological adaptations through complex molecular regulation across multiple biological systems. While plasma-based proteomics has substantially advanced our understanding of exercise-associated molecular changes, the proteomic responses of mucosal biofluids, including tear fluid and nasal secretions, remain poorly characterized. A multi-biofluid proteomic approach may provide a more comprehensive characterization of molecular alterations associated with acute exercise.

**Methods:**

Paired samples of plasma, tear fluid, and nasal secretions were collected from five young, healthy female participants before and after acute aerobic exercise and analyzed using data-independent acquisition (DIA) liquid chromatography-tandem mass spectrometry (LC–MS/MS). Proteomic profiles across the three biofluids were characterized, and joint mixed-effects modeling was performed to identify proteins exhibiting common or biofluid-specific exercise-associated responses. Functional enrichment analyses were conducted to characterize the functional categories associated with upregulated and downregulated proteins in each biofluid.

**Results:**

Highly abundant proteins shared across the three biofluids showed enrichment in immune-related processes, inflammatory responses, and protease regulation. Joint mixed-effects analysis identified proteins exhibiting common exercise-associated responses across biofluids, which were primarily enriched in translation-related proteins, including ribosomal proteins, as well as proteins showing biofluid-specific exercise-associated responses. Acute exercise induced differential proteomic alterations across the three biofluids, with differentially abundant proteins showing distinct functional enrichment patterns among the three biofluids.

**Conclusion:**

This study provides a multi-biofluid DIA proteomic characterization of acute exercise-associated molecular alterations. The findings demonstrate that exercise-induced proteomic changes differ across plasma, tear fluid, and nasal secretions, highlighting the value of integrating multiple biofluids to characterize molecular responses associated with exercise. These results provide a foundation for future studies involving larger cohorts and targeted validation.

## 1 Introduction

Exercise is a fundamental health-promoting intervention that induces broad physiological adaptations across multiple biological systems (Qiu et al., 2023; Ahn et al., 2025). Regular exercise contributes to metabolic homeostasis, cardiovascular function, and immune regulation, thereby supporting the maintenance of physiological balance and reducing the risk of chronic diseases (Nieman and Wentz, 2019; Amare et al., 2024; Esteves and Stanford, 2024; Jin et al., 2024; Ha et al., 2025; Mi et al., 2025). These diverse adaptations are mediated by complex molecular networks involving coordinated changes across multiple biological compartments and physiological processes. Biological fluids represent important sources of molecular information that reflect physiological alterations induced by exercise. Therefore, systematic characterization of molecular alterations across diverse biological fluids has become increasingly important for elucidating the biological mechanisms underlying exercise adaptation.

At the proteomic level, the research paradigm for investigating the molecular mechanisms underlying the health benefits of exercise is undergoing significant evolution (Amar et al., 2024; MoTrPAC Study Group et al., 2024). Early studies primarily focused on the proteomic profiling of individual core tissues or biofluids, such as plasma, skeletal muscle, and liver (Hoffman et al., 2015; Murgia et al., 2017; De Nardo et al., 2022). Plasma, as the central carrier for systemic signal transduction, has become the most extensively studied biological fluid, providing crucial insights into exercise-induced changes in metabolic, inflammatory, and endocrine signaling pathways. With the advancement of systems biology, exercise proteomics is shifting from isolated analyses towards the synchronous, integrated analysis of multiple tissues and biofluids to construct a more comprehensive exercise-responsive molecular network (Shen et al., 2024; Yu et al., 2021; Guo et al., 2025).

Despite substantial progress in related research, current exercise proteomics studies remain largely focused on plasma. While plasma-based research has substantially advanced our understanding of systemic molecular responses to exercise, other biologically informative biofluids have received limited attention. Consequently, the exercise-associated molecular alterations captured by diverse biofluids remain poorly characterized.

Tear fluid and nasal secretions, as integral components of the human mucosal immune barrier, possess unique physiological and investigative value. Tear fluid not only lubricates and protects the ocular surface but also defends against pathogen invasion (de Souza et al., 2006; Rantamäki et al., 2011; Pflugfelder and Stern, 2020), whereas nasal secretions contain defensins, surfactant proteins, and secretory immunoglobulin A, supporting frontline immune defense and airway conditioning in the upper respiratory tract (Lane, 2009; Tomazic et al., 2020). However, proteomic investigations of these biofluids have largely focused on local pathological conditions, such as dry eye disease and allergic rhinitis (Wang et al., 2011; Kannan et al., 2023), and their dynamic molecular responses to exercise remain largely unexplored. Although a few studies have examined exercise-related protein regulation in conventional secretions such as saliva and urine (Lindsay and Costello, 2017; Ong et al., 2023), tear fluid and nasal secretions have not been systematically evaluated within the context of exercise physiology.

The emerging field of multi-biofluid integration still lacks a systematic comparative analysis between circulatory plasma and local mucosal secretions (Kurgan et al., 2025). Therefore, this study aimed to systematically compare the proteomic profiles of tear fluid, nasal secretions, and plasma following exercise. By identifying shared and biofluid-specific high-abundance proteins, we sought to elucidate common systemic responses and local mucosal adaptations to exercise. This multi-biofluid integrative approach provides new insights into the coordinated molecular regulation of exercise across physiological compartments and offers a foundation for developing non-invasive strategies for monitoring exercise-induced health adaptations.

## 2 Materials and methods

### 2.1 Study design

Five healthy female participants, aged 23–27 years, with no history of exercise addiction or long-term professional training, were enrolled in this study. Potential influences of menstrual cycle-related hormonal fluctuations were considered, and all sample collections and exercise interventions were scheduled outside the menstrual phase. To minimize metabolic state variations caused by dietary differences, all participants were required to strictly adhere to a standardized diet for three days prior to the exercise intervention. This protocol prohibited the consumption of alcohol, coffee, strong tea, caffeine-containing beverages, as well as high-sugar, high-fat, and spicy stimulant foods.

A moderate-intensity aerobic exercise protocol (treadmill running) was uniformly administered. Exercise intensity was precisely controlled using individual maximum heart rate (HRmax), calculated according to the Tanaka formula: HRmax = 208 – (0.7 × age) (Tanaka et al., 2001). The target intensity range was set at 60%–80% of HRmax. The exercise session began with a 5-minute warm-up on the treadmill at 4 km/h, followed by 30 minutes of continuous running while maintaining the target heart rate zone. Heart rate was monitored in real time throughout the session using a Polar H10 chest strap to ensure exercise intensity remained within the prescribed range. Perceived exertion was also recorded using the Borg 6–20 rating of perceived exertion (RPE) scale; the mean score across participants was 12.4 (**Supplementary File 1**). Blood, tear fluid, and nasal secretion samples were collected immediately before and after the exercise session.

All exercise interventions and sample collections were conducted within a standardized morning time window. Pre-exercise blood, tear fluid, and nasal secretion samples were collected between 8:30 and 9:00 AM, and post-exercise samples were collected within 10 minutes after completion of the exercise protocol (between 10:00 and 11:00 AM).

The study protocol was approved by the Ethics Committee of the Wuhan Sports University (Approval No. 2025089), and written informed consent was obtained from all participants.

### 2.2 Plasma samples collection and processing

Five milliliters of cubital venous blood were collected and transferred into EDTA-containing vacuum blood collection tubes. After collection, the tubes were gently inverted several times to ensure adequate mixing and immediately placed on ice. Within 30 minutes of collection, the samples were centrifuged at 4°C and 4000 rpm for 5 minutes to separate the cellular components. Following centrifugation, the upper plasma layer was carefully aspirated using a pipette. The collected plasma was then aliquoted into cryovials and stored at -80°C until subsequent proteomic analysis. For sample processing, the plasma was thawed at 4 °C. For each sample, 2 µL of plasma was aliquoted and mixed with 48 µL of UA lysis buffer, followed by incubation at 37°C for 1 hour. The mixture was then subjected to ultrasonication for 2 minutes and centrifuged at 16,000 × g for 20 minutes at 4°C. The resulting supernatant was collected for subsequent analysis.

### 2.3 Tear fluid samples collection and processing

Tear fluid samples were collected bilaterally using Schirmer test strips without local anesthesia, and sterile strips (5 mm wide) were placed at the outer one-third of the lower conjunctival sac until fully saturated (Willcox et al., 2017). The same collection procedure was applied consistently at both pre- and post-exercise assessments. Tear-saturated strips were immediately transferred to a pre-cooled container and then stored at −80°C for subsequent analysis. For processing, samples were thawed at 4°C. Each test strip was then transferred to a microcentrifuge tube, and 400 μL of ice-cold methanol-water solution (4:1, v/v) was added. The mixture was subjected to ultrasonication in an ice bath for 30 minutes, followed by incubation at −20°C for 30 minutes. After centrifugation at 16,000 × g for 20 minutes at 4°C, the supernatant was collected for subsequent mass spectrometry analysis.

### 2.4 Nasal secretion samples collection and processing

A volume of 2.5-5.0 mL of physiological saline (0.9% NaCl; N24100610; Kelun Pharmaceutical, Chengdu, China), pre-warmed to 37°C, was instilled into each nostril with the participant seated, neck extended backward to 30 degrees from the horizontal plane, nasal cavities upward, breath held, and mouth slightly open, and retained for approximately 10 seconds. The same standardized lavage procedure was applied at both pre- and post-exercise assessments. The fluid was then expelled from the nostrils and collected into pre-chilled tubes. All samples were stored at −80°C until analysis. Prior to analysis, each sample was lyophilized. An appropriate volume of SDT lysis buffer was added to the lyophilized material, which was then transferred to a microcentrifuge tube. The mixture was heated in a boiling water bath for 3 minutes, followed by ultrasonication for 2 minutes. Subsequently, it was centrifuged at 16,000 × g for 20 minutes at 4°C, and the resulting supernatant was collected for further processing.

### 2.5 Proteomic analysis by data-independent acquisition mass spectrometry

Quantitative proteomic analysis was performed using data-independent acquisition (DIA). Following protein extraction, the total protein concentration of each sample was quantified using the bicinchoninic acid (BCA) assay, and equal amounts of total protein from each sample were subjected to enzymatic digestion. Tear-fluid and nasal-secretion proteins were subjected to filter-aided sample preparation (FASP), with reduction using DTT, alkylation using IAA, and digestion with 6 μg trypsin at 37°C for 16–18 h. For plasma samples, low-abundance proteins were enriched prior to digestion, followed by reduction with dithiothreitol (DTT) (final concentration, 100 mM), alkylation with iodoacetamide (IAA) (final concentration, 50 mM), and digestion with 8 μg trypsin at 37°C for 20 h. Following digestion, peptides were desalted using C18 cartridges, dried, reconstituted in 0.1% formic acid, and peptide concentrations were measured prior to LC-MS/MS analysis. Proteins extracted from samples were digested with trypsin.

The resulting peptides were separated using a Vanquish Neo ultra-high-performance liquid chromatography (UHPLC) system (Thermo Scientific, Waltham, MA, USA) equipped with a PepMap Neo C18 trap column (5 μm, 300 μm × 5 mm) and a μPAC Neo High Throughput analytical column. Mobile phase A consisted of 0.1% formic acid in water, whereas mobile phase B consisted of 0.1% formic acid in 80% acetonitrile. The chromatographic gradient was programmed as follows: 4–6% B from 0 to 0.1 min, 6–12% B from 0.1 to 1.1 min, 12–22.5% B from 1.1 to 4.3 min, 22.5–45% B from 4.3 to 6.1 min, followed by 99% B until 8 min.

The separated peptides were subsequently analyzed by LC-MS/MS in DIA mode using an Orbitrap Astral mass spectrometer (Thermo Scientific). The precursor-ion scan range was 380–980 m/z with an MS1 resolution of 240,000, an AGC target of 500%, and a maximum injection time of 3 ms. DIA MS/MS acquisition was performed using a 2 Th isolation window, an MS2 resolution of 80,000, an AGC target of 500%, a maximum injection time of 3 ms, HCD fragmentation, a normalized collision energy of 25%, an RF-lens setting of 40%, and a cycle time of 0.6 s.

During DIA acquisition, the full mass scan range was divided into consecutive windows, and all precursor ions within each window were cyclically fragmented to unbiasedly acquire fragment ion information for all detectable peptides. All raw MS data were processed, searched, and quantified using the DIA-NN software (version 1.8). Trypsin was specified as the proteolytic enzyme, allowing a maximum of one missed cleavage. Carbamidomethylation of cysteine was specified as a fixed modification, whereas oxidation of methionine and protein N-terminal acetylation were specified as variable modifications. Tear-fluid and nasal-secretion data were searched against the UniProtKB *Homo sapiens* (Human) [9606] database (uniprotkb-Homo sapiens (Human) [9606]-205092-20250414.fasta), whereas plasma data were searched against the reviewed Swiss-Prot *Homo sapiens* (Human) [9606] database (swissprot-Homo sapiens (Human) [9606]-reviewed-20418-20250414.fasta). Both databases were downloaded on April 14, 2025. A target–reverse database-search strategy was applied, and the false discovery rate (FDR) was controlled at 1% at both the peptide-spectrum match (PSM) and protein levels. Protein inference was performed using the built-in maximum parsimony algorithm implemented in DIA-NN. Protein quantification was based on the integrated peak areas of high-confidence peptide fragment ions.

### 2.6 Data preprocessing and differential abundance analysis

All statistical analyses were performed using R software (version 4.6.1). Protein expression levels were quantified based on mass spectrometry (MS) intensity values. For each biofluid, proteins without an assigned gene symbol were removed. When multiple protein groups mapped to the same gene symbol, redundant entries were consolidated by retaining the representative entry with the highest quantitative completeness, followed by peptide evidence (unique peptide number). The remaining proteins were retained if valid quantitative values were available in at least 50% of samples in either the pre-exercise or post-exercise condition.

For differential abundance analysis within each biofluid, MS intensity values were log_2_-transformed and subsequently normalized using sample-wise median normalization. Missing values were not imputed for differential abundance analysis. Differential protein abundance between the pre- and post-exercise conditions was assessed using the limma package with a paired linear model. Participant identity and exercise condition were included in the design matrix (~ Subject + Condition) to account for repeated measurements from the same participants. Proteins were required to have valid quantitative measurements in at least three complete pre–post pairs. Empirical Bayes moderation was applied using the eBayes function with trend and robust estimation. Proteins meeting the criteria of |log_2_FC| > log_2_(1.5) and a nominal *P*-value < 0.05 were identified as differentially abundant proteins, while BH-adjusted P-values were additionally calculated and reported.

### 2.7 Cross-biofluid comparison, identification of shared high-abundance proteins, and PPI network analysis

For cross-biofluid comparisons, protein abundances were normalized separately within each biofluid. Baseline abundance profiles were compared across plasma, tear fluid, and nasal secretions, and proteins shared among all three biofluids were identified. Proteins within the top 10% of baseline abundance in each biofluid were defined as high-abundance proteins, and proteins meeting this criterion simultaneously in all three biofluids were considered shared high-abundance proteins.

A protein-protein interaction (PPI) network was constructed for the shared high-abundance proteins using the STRING database (version 12.0) with *Homo sapiens* selected as the organism. The proteins were uploaded as multiple proteins, and only high-confidence interactions (combined score > 0.700) were retained for network construction. The combined score integrates multiple evidence channels, including experimentally determined interactions, curated database annotations, co-expression, gene neighborhood, gene fusion, and text mining. Proteins without any high-confidence interaction partner (isolated nodes) were excluded from the final network. The PPI network was visualized using Cytoscape software, and proteins were ranked according to degree centrality to identify highly connected nodes.

### 2.8 Joint mixed-effects analysis of shared proteins

For proteins shared across all three biofluids, a joint linear mixed-effects analysis was performed to distinguish common exercise responses from biofluid-specific responses. Exercise condition and biofluid type were included as fixed effects, and participant was included as a random intercept. For each shared protein, an additive model (Intensity ~ Condition + Biofluid + (1|Subject)) was compared with a model including the condition × biofluid interaction (Intensity ~ Condition * Biofluid + (1|Subject)) using a likelihood-ratio test. BH correction was applied to both the interaction and overall condition-effect P-values. Proteins with a significant condition × biofluid interaction (BH-adjusted P-value < 0.05) were classified as biofluid-specific responses, whereas proteins without a significant interaction but with a significant overall condition effect (BH-adjusted P-value < 0.05) were classified as common exercise responses. Proteins with unstable interaction-model fits were excluded from response classification.

### 2.9 Functional enrichment analysis

Gene Ontology (GO) functional enrichment analysis and Kyoto Encyclopedia of Genes and Genomes (KEGG) pathway enrichment analysis were performed using the clusterProfiler and org.Hs.eg.db packages in R. Shared high-abundance proteins across the three biofluids were analyzed by GO enrichment with BH-adjusted significance thresholds. For proteins uniquely detected in each biofluid, GO and KEGG enrichment analyses were conducted using these proteins as the foreground set and all proteins identified across the three biofluids as the background reference set. For differentially abundant proteins within each biofluid, KEGG enrichment was performed separately for upregulated and downregulated proteins, with proteins entering the differential abundance analysis used as the background set. Pathways with BH-adjusted P-value < 0.05 and q < 0.05 were considered significantly enriched; for datasets in which no terms or pathways passed the multiple-testing correction threshold, nominal P-value < 0.05 enrichment results were reported as exploratory findings.

### 2.10 Partial least squares-discriminant analysis

For candidate feature proteins, partial least squares-discriminant analysis (PLS-DA) was employed to calculate variable importance in projection (VIP) scores. PLS-DA was performed separately for each biofluid using the differentially abundant proteins as input features. Missing values in the PLS-DA input matrix were imputed using k-nearest-neighbor imputation (k = 3), and two latent components were fitted. Model performance was evaluated using leave-one-out cross-validation (LOOCV), including the overall classification error rate and balanced error rate (BER), and model significance was further assessed using 500 permutation tests. The top 25 proteins ranked by VIP score from each biofluid group were selected as candidate feature proteins.

## 3 Results

### 3.1 Profiling and comparison of quantified proteomes across three biofluids

Samples of plasma, tear fluid, and nasal secretions were collected from five healthy female participants immediately before and after a standardized exercise intervention. Participant characteristics are summarized in **Supplementary Table S1**. After applying the predefined filtering criteria, a total of 2274 unique proteins were identified in plasma, 3258 in tear fluid, and 3542 in nasal secretions (**Supplementary Table S2**). Overall, 4,997 non-redundant proteins were identified across the three biofluids. Among these, 1200 proteins were commonly detected across all three biofluids, whereas 673, 702, and 745 proteins were uniquely detected in plasma, tear fluid, and nasal secretions, respectively (**Figure 1A**). The distribution of identified proteins among plasma, tear fluid, and nasal secretions, including shared and biofluid-specific proteins, is summarized in **Supplementary Table S3**. The normalized protein abundance patterns across the three biofluids are presented in **Figure 1B**. Based on relative abundance within each biofluid, 54 of these shared proteins were identified as highly abundant across all three biofluids.

**Figure 1:**
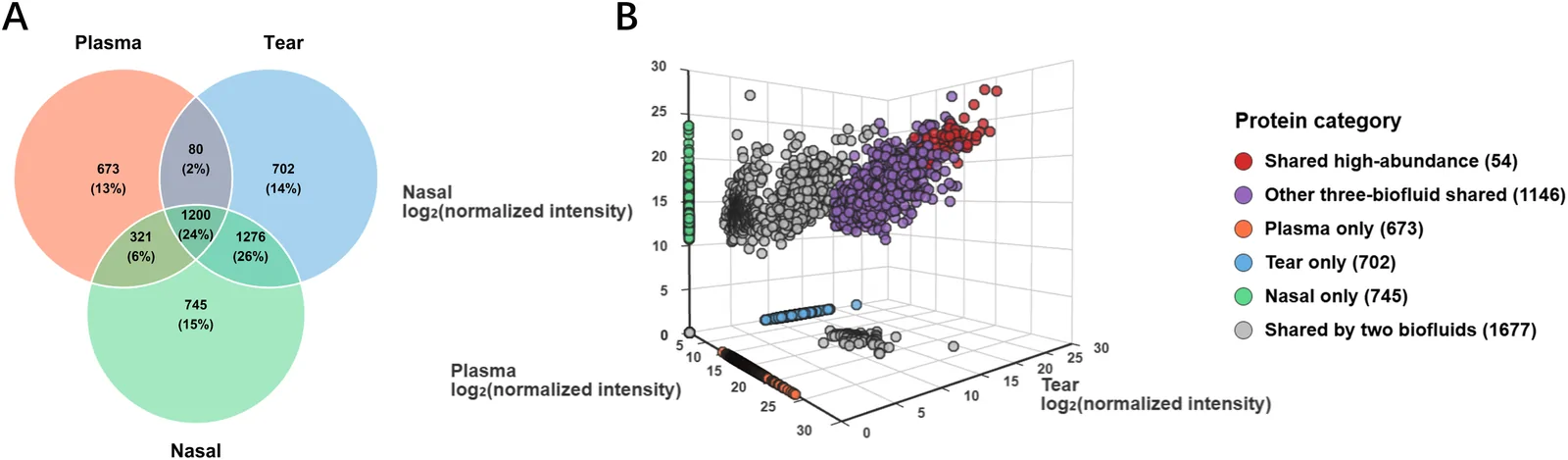
Overlap and expression profiles of proteins detected across plasma, tear fluid and nasal secretions. **(A)** Venn diagram showing the overlap of quantified proteins among plasma, tear fluid and nasal secretions. **(B)** The three-dimensional scatter plot showing the log_2_-transformed median protein abundance profiles across plasma, tear fluid, and nasal secretions. Proteins that were highly abundant in all three biofluids are indicated in red. Other proteins shared among the three biofluids are shown in purple, whereas proteins detected exclusively in plasma, tear fluid, or nasal secretions are indicated in orange, blue, and green, respectively. Proteins shared by two biofluids are shown in gray.

### 3.2 Comparative and integrated analysis of high abundance proteins across three biofluids

Among the highly abundant proteins shared across the three biofluids, 30 proteins were retained for PPI network construction after applying the interaction confidence threshold (**Figure 2A**). Several proteins with high degree values were identified, including ALB, APOA1, SERPINA1, C3, CLU, and TTR. In addition, GO enrichment analysis revealed that this network was associated with immune-related biological processes, including defense response, immune system regulation, and inflammatory responses. These proteins were mainly localized to secretory vesicles, vesicle lumen, and other extracellular vesicle-related compartments. Molecular function analysis further indicated enrichment in protease inhibitor activity, peptidase regulator activity, and molecular carrier functions (**Figure 2B**). Biofluid-specific proteins were further analyzed by GO and KEGG enrichment analyses. Proteins unique to each biofluid (i.e., detected exclusively in a single biofluid) were used as the foreground set and the 4,997 proteins commonly detected across all three biofluids used as the reference background. Plasma - specific proteins were mainly enriched in immune-related functions, extracellular matrix organization, and receptor-mediated signaling. Representative GO terms included leukocyte migration, G protein-coupled receptor signaling, extracellular matrix components, and receptor ligand activity, while KEGG analysis highlighted cytokine–cytokine receptor interaction, chemokine signaling pathway, and ECM–receptor interaction (**Supplementary Figures S1A, D**). Tear fluid specific proteins showed predominant enrichment in protein modification-related processes. GO analysis identified terms related to post-translational protein modification, protein ubiquitination, and ubiquitin-related transferase activity, with KEGG pathways including NOD-like receptor signaling, TNF signaling, and ubiquitin-mediated proteolysis (**Supplementary Figures S1B, E**). Nasal secretions - specific proteins exhibited broad functional enrichment. Enriched GO terms included receptor-activity annotations, alongside mRNA-processing and spliceosome-related components, while exploratory KEGG analysis based on nominal *P*-values identified enrichment of cytokine–cytokine receptor interaction and cell-adhesion molecule interaction, along with spliceosome-related pathways (**Supplementary Figures S1C, F**). Detailed GO and KEGG enrichment results for plasma, tear fluid, and nasal secretion specific proteins are provided in **Supplementary Table S4**.

**Figure 2:**
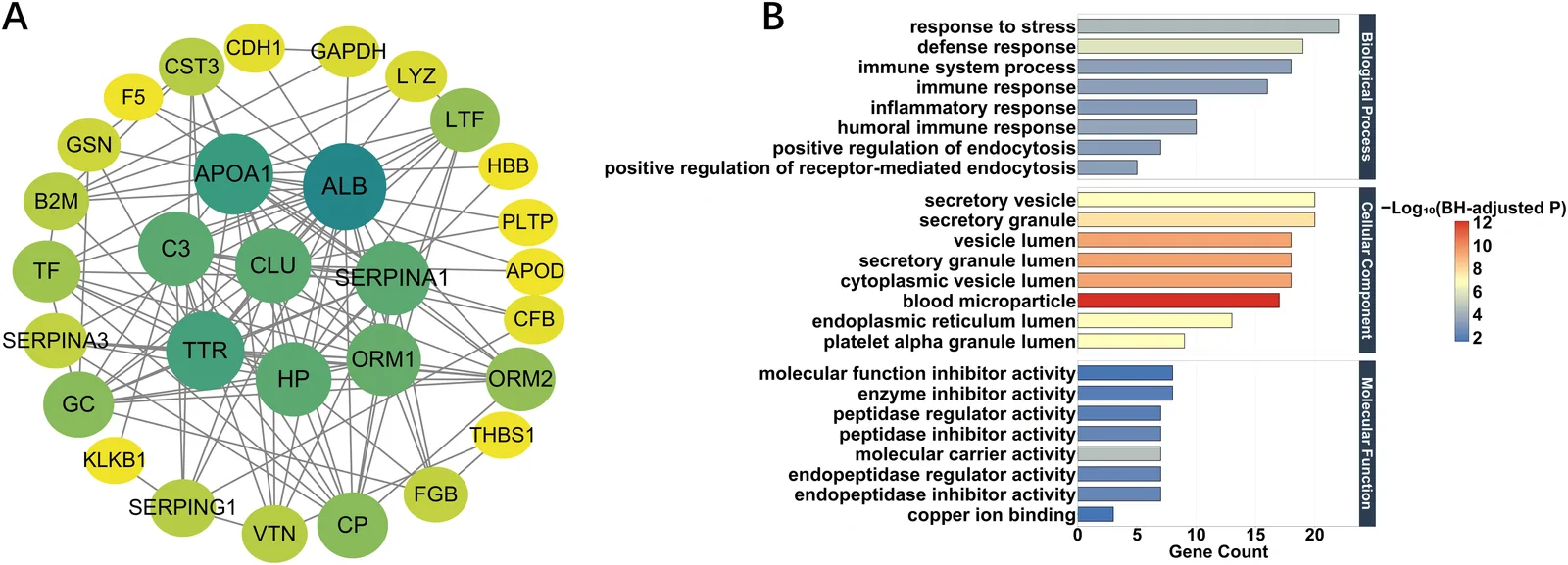
PPI network and GO enrichment analysis of highly abundant shared proteins across plasma, tear fluid, and nasal Secretions. **(A)** A network displaying the interactions among highly abundant shared proteins identified across plasma, tear fluid and nasal secretions. **(B)** The bar chart shows the significantly enriched GO terms for the genes corresponding to the highly abundant shared protein.

### 3.3 Joint mixed-effects analysis reveals common and biofluid-specific exercise-responsive proteins across plasma, tear fluid, and nasal secretions

To further characterize exercise-associated proteomic responses across biofluids, a joint linear mixed-effects model was applied to the 1,200 proteins commonly detected in plasma, tear fluid, and nasal secretions. Exercise condition and biofluid were included as fixed effects, while participant was modeled as a random effect. Likelihood-ratio tests comparing models with and without the exercise-by-biofluid interaction were performed to distinguish proteins showing consistent exercise-associated responses across biofluids from those exhibiting biofluid-dependent responses. Among these shared proteins, 13 proteins showed a significant main effect of exercise without a significant interaction effect, indicating a comparable response pattern across the three biofluids (**Figure 3A**). These proteins showed predominantly increased responses after exercise, with several translation-associated proteins, including RPL30, RPL14, RPL22, and RPS16, exhibiting relatively larger effect estimates.

**Figure 3:**
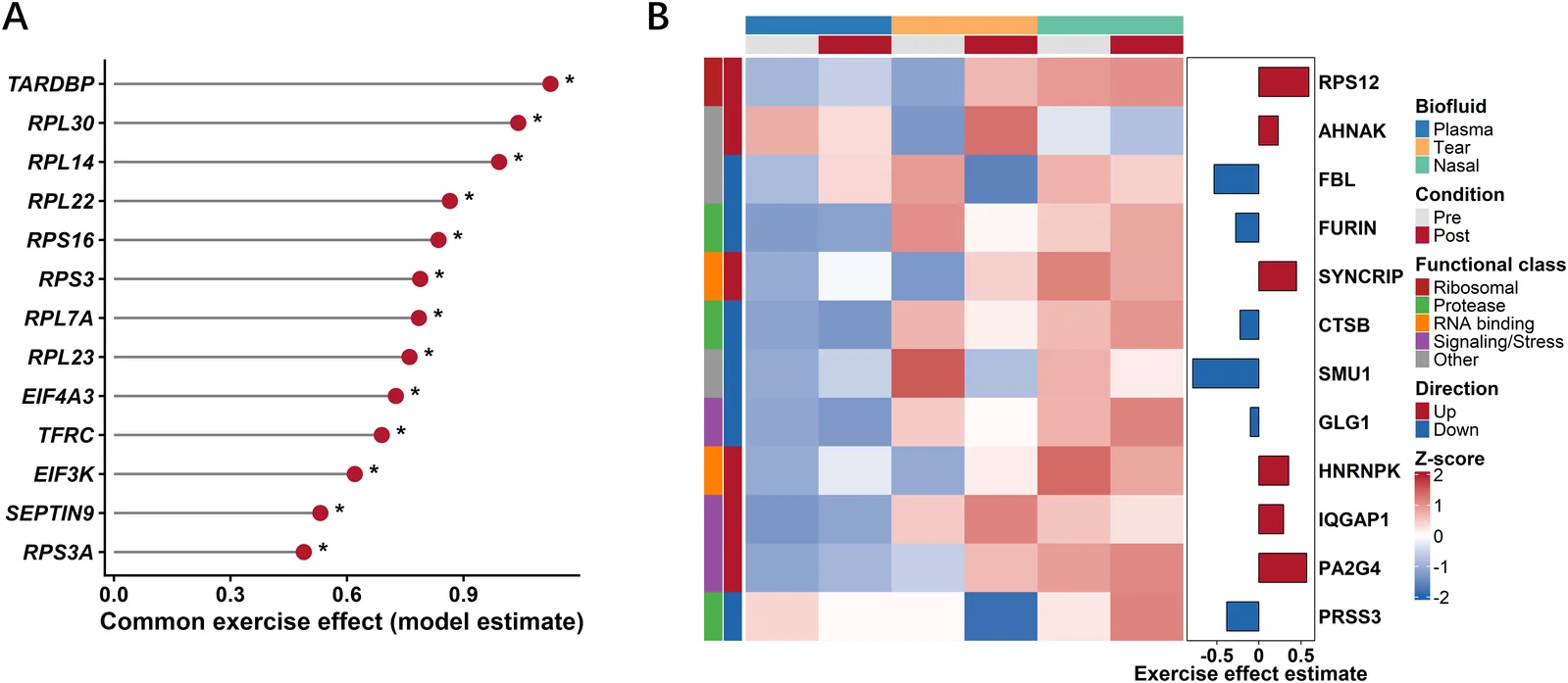
Joint mixed-effects analysis of shared proteins across plasma, tear fluid, and nasal secretions. **(A)** Proteins with significant common exercise effects identified by the joint mixed-effects model. **(B)** Heatmap displaying exercise-associated effect estimates of shared proteins across the three biofluids.

In contrast, 12 proteins displayed significant exercise-by-biofluid interactions, indicating that exercise-induced alterations in these proteins were biofluid-dependent (**Figure 3B**). These proteins exhibited divergent response profiles across plasma, tear fluid, and nasal secretions. For instance, FURIN, CTSB, and SYNCRIP showed contrasting post-exercise abundance patterns between plasma and tear fluid, while FBL and SMU1 displayed distinct response profiles in nasal secretions relative to the other biofluids. Moreover, signaling- and stress-related proteins, including PA2G4 and IQGAP1, exhibited biofluid-specific differences in the magnitude of exercise-associated changes. The full results of the joint linear mixed-effects model are provided in **Supplementary Table S5**. Collectively, these findings suggest that exercise-induced proteomic regulation is not uniform across biological fluids.

### 3.4 Characterization of KEGG pathway enrichment in the multi-biofluid proteome following acute exercise

To characterize the functional consequences of acute exercise-induced proteomic alterations across different biological fluids, differential protein abundance patterns and pathway enrichment profiles were systematically evaluated in plasma, tear fluid, and nasal secretions. Following acute exercise, plasma exhibited 148 significantly upregulated and 89 significantly downregulated proteins, whereas tear fluid showed a broader proteomic response with 525 upregulated and 235 downregulated proteins. Nasal secretions demonstrated a comparatively smaller but distinct response, with 57 upregulated and 116 downregulated proteins (**Figures 4A–C**).

**Figure 4:**
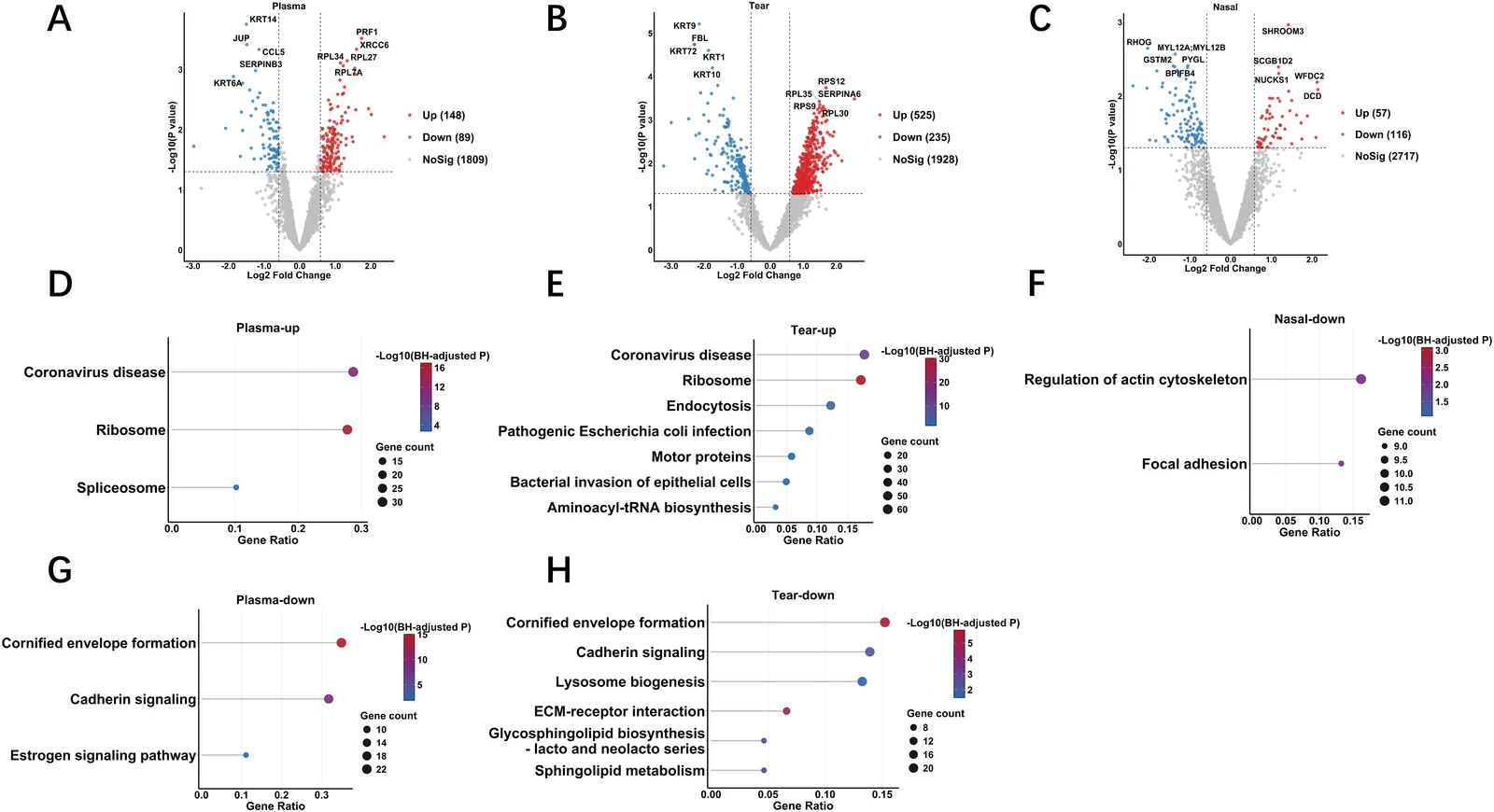
Functional annotation of differentially abundant proteins in plasma, tear fluid, and nasal secretions before and after exercise. The figure is organized into three columns corresponding to each biofluid: plasma (left column: **A, D, G**), tear fluid (middle column: **B, E, H**), and nasal secretions (right column: **C, F**). **(A–C)** Volcano plots showing differentially abundant proteins identified between pre- and post-exercise conditions in each biofluid. **(D, E)** KEGG pathway enrichment analysis of upregulated proteins in plasma and tear fluid. **(F–H)** KEGG pathway enrichment analysis of downregulated proteins in nasal secretions, plasma, and tear fluid, respectively.

KEGG enrichment analysis was performed separately for upregulated and downregulated proteins in each of the three biofluids. Significantly altered proteins were used as the foreground set, while all quantified proteins retained after preprocessing within the corresponding biofluid were used as the background reference set. Among upregulated proteins, plasma showed enrichment of pathways mainly associated with protein synthesis and RNA processing, including ribosome, spliceosome, and Coronavirus disease pathway annotation (**Figure 4D**). In the tear fluid significantly upregulated protein set, enrichment was observed in ribosome-related and pathogen-associated pathways including Coronavirus disease, ribosome, and endocytosis (**Figure 4E**). Given that the Coronavirus disease pathway annotation was enriched in both plasma and tear fluid, the contributing genes within this annotation were further characterized to clarify its biological context. The majority of contributing genes were ribosomal protein genes, with 30 of 31 genes in plasma and 58 of 61 genes in tear fluid belonging to the RPL and RPS families, accounting for 96.8% and 95.1% of the annotated genes, respectively (**Supplementary Table S6**). In nasal secretions, pathways enrichment based on nominal *P*-value < 0.05 was considered as exploratory interpretation, revealing enrichment of pathways related to neurodegeneration-associated annotations, including Alzheimer’s disease, Parkinson’s disease, Huntington’s disease, Amyotrophic lateral sclerosis, and Pathways of neurodegeneration–multiple diseases (**Supplementary Figure S2**). Examination of pathway-contributing proteins revealed substantial overlap among these disease-related annotations, which were mainly driven by a limited number of shared proteins, including MT-CO2, VDAC1, PSMD4, NDUFS3, SNCA, and TARDBP (**Supplementary Table S6**).

Conversely, in the plasma significantly downregulated protein set, enriched pathways included cornified envelope formation, cadherin signaling, and estrogen signaling pathway, which are predominantly associated with epithelial structural and cell interaction-related processes (**Figure 4G**). In the tear fluid significantly downregulated protein set, enriched pathways included cornified envelope formation, lysosome biogenesis, ECM–receptor interaction, and sphingolipid metabolism (**Figure 4H**). Similarly, in the nasal secretions significantly downregulated protein set, enriched pathways included regulation of actin cytoskeleton and focal adhesion (**Figure 4F**).

### 3.5 PLS-DA identifies distinct exercise-responsive protein signatures across biofluids

Partial least squares-discriminant analysis (PLS-DA) identified proteins with high discriminatory capacity between pre- and post-exercise states. Proteins with VIP > 1 and significant differential abundance were retained as candidate feature proteins (**Supplementary Table S7**), and the top 25 ranked proteins for each biofluid are shown in **Figures 5A–C**. The heatmap illustrates the Z-score standardized expression patterns of the selected 75 candidate feature proteins (the top 25 from each biofluid) across pre-exercise and post-exercise samples (**Figure 5D**). The distinct abundance patterns and the absence of overlap among the top 25 VIP proteins across plasma, tear fluid, and nasal secretions indicate that the proteins contributing most strongly to pre- and post-exercise sample discrimination were largely biofluid-specific rather than shared across compartments. These findings provide an exploratory characterization of protein features contributing to the discrimination between pre- and post-exercise samples within each biofluid.

**Figure 5:**
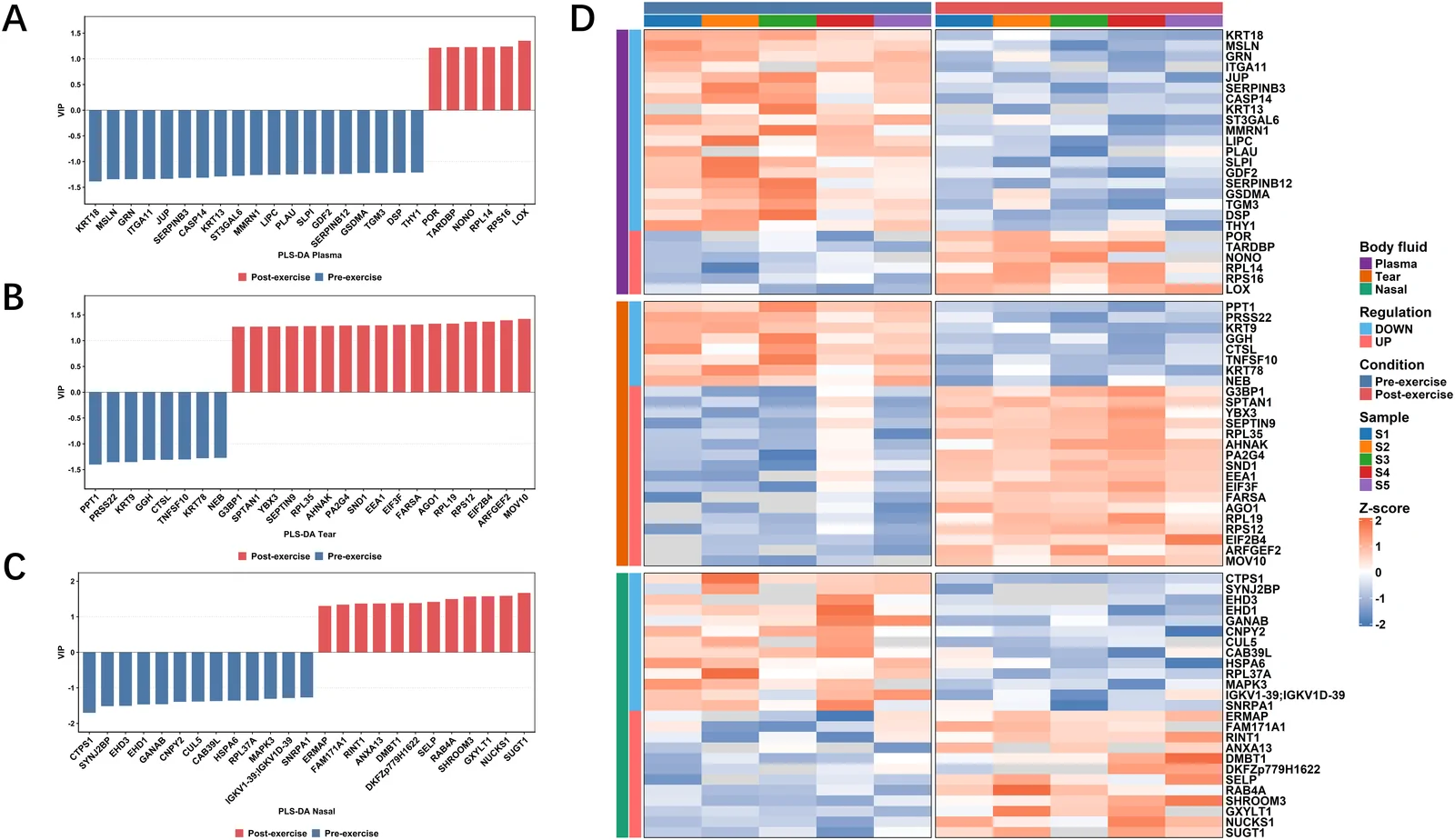
VIP scores and expression heatmap of PLS-DA-derived discriminatory proteins across three biofluids. **(A)** Top 25 proteins ranked by VIP scores in plasma. **(B)** Top 25 proteins ranked by VIP scores in tear fluid. **(C)** Top 25 proteins ranked by VIP scores in nasal secretions. **(D)** Expression heatmap of the discriminatory proteins identified from the three biofluids.

## 4 Discussion

This study provides a comprehensive multi-biofluid proteomic characterization of acute exercise-associated molecular alterations by integrating paired samples of plasma, tear fluid, and nasal secretions obtained from the same subjects before and after acute exercise, using data-independent acquisition liquid chromatography-tandem mass spectrometry (LC–MS/MS). By simultaneously performing full proteome profiling of these three circulating and mucosal biofluids, our research provides insights into how different biological compartments capture distinct aspects of exercise-associated proteomic changes and helps elucidate the integrated physiological response network to acute exercise from a combined multi-biofluid perspective.

Previous research has demonstrated that different biofluids exhibit characteristic proteomic profiles influenced by their biological origins and physiological environments. Plasma proteins comprise abundant circulating components, such as albumin, lipoproteins, globulins, and complement proteins, together with lower-abundance proteins originating from peripheral tissues (Anderson and Anderson, 2002; Bankier et al., 2025). Tear fluid plays a key role in physiological functions such as immune defense, antibacterial activity, lubrication, and nutrient supply (McDermott, 2013; Pflugfelder and Stern, 2020). The most abundant proteins in tear fluid have been identified as members of the immunoglobulin, and complement families (Ponzini et al., 2021). Nasal secretion proteins mainly contribute to mucosal defense, with mucins increasing secretion viscosity, secretory immunoglobulin A (sIgA) mediating immune protection, and lysozyme and lactoferrin proteins providing antibacterial effect (Raphael et al., 1989; Martínez‐Antón et al., 2006; Li et al., 2020). Although biofluids differ in their overall protein composition, our study identified a large number of proteins commonly detected across plasma, tear fluid, and nasal secretions. Among these shared proteins, highly abundant proteins included albumin (ALB), apolipoprotein A1 (APOA1), complement component 3 (C3), clusterin (CLU), serpin family A member 1 (SERPINA1), and transthyretin (TTR), which are major extracellular proteins involved in protein transport, complement regulation, protease control, and molecular homeostasis. Consistent with these characteristics, Gene Ontology enrichment analysis of highly abundant shared proteins revealed associations with immune-related processes, secretory vesicle-associated compartments, and molecular functions related to protease regulation and carrier activity. These findings suggest that the common high-abundance proteins across different biofluids are primarily associated with conserved extracellular functions, including immune-related processes, protein regulation, and molecular transport, reflecting shared biological characteristics of circulating and mucosal biofluids.

For proteins uniquely detected in each of the three biofluids, large-scale plasma proteome studies have shown that circulating proteins are prominently involved in extracellular signaling and immune regulation, with receptor-mediated pathways and cytokine signaling among the most represented functional categories (Deutsch et al., 2021; Sun et al., 2023). In tear fluid, proteomic analyses have repeatedly identified a high proportion of proteases, protease regulators, and post-translationally modified proteins, indicating active protein turnover and post-translational regulation at the ocular surface (Ponzini et al., 2021). For nasal secretions, airway mucosal fluid is known to harbor abundant epithelial-derived and immune-related proteins mediating local innate defense (Tomazic et al., 2020). In the present study, nasal secretion-unique proteins were also enriched for RNA processing and splicing-related functions, a pattern that may reflect epithelial-derived or vesicle-associated components in upper airway mucosal fluid. Collectively, these findings indicate that proteins uniquely detected in each biofluid are associated with distinct functional categories, reflecting differences in their proteomic composition.

A noteworthy observation was that proteins significantly upregulated after exercise in both plasma and tear fluid were enriched in Coronavirus disease and ribosome-related pathways, which may initially appear unexpected. However, Reimand et al. have indicated that disease-associated annotations in KEGG and other pathway enrichment analyses can be potentially misleading (Reimand et al., 2019), as they frequently encompass broadly expressed housekeeping proteins, such as ribosomal and mitochondrial proteins. Further inspection of the contributing genes revealed that the Coronavirus disease annotation was predominantly composed of ribosomal protein genes, indicating that this pathway annotation primarily reflected ribosome-associated translational components rather than a disease-specific biological response. Similarly, examination of the neurodegeneration-related pathways enriched among upregulated proteins in nasal secretions demonstrated that the contributing genes were mainly involved in mitochondrial function, proteostasis, and cellular regulatory processes. Furthermore, the enrichment of cornified envelope formation among downregulated proteins in plasma and tear fluid warrants cautious interpretation, as keratin contamination represents a common challenge in mass spectrometry-based proteomics (Mellacheruvu et al., 2013).

The joint mixed-effects analysis further revealed the exercise-associated response patterns of commonly detected proteins across biofluid compartments. Although many proteins were detected across plasma, tear fluid, and nasal secretions, their shared presence did not necessarily indicate identical responses to acute exercise. By incorporating exercise condition and biofluid as fixed effects while accounting for inter-individual variability, this analysis revealed that ribosomal proteins exhibited uniform post-exercise responses across the three biofluids, consistent with the enrichment of upregulated proteins in the biofluids after exercise, suggesting that translation-associated molecular features were coordinately upregulated across different biological compartments. In contrast, proteins exhibiting significant exercise-by-biofluid interactions, such as FURIN, CTSB, and SYNCRIP, displayed divergent response patterns among plasma, tear fluid, and nasal secretions. These differences indicate that individual proteins may exhibit distinct exercise-associated responses across biofluids, emphasizing the importance of considering the biological context of each sampling compartment when interpreting multi-biofluid proteomic responses.

PLS-DA identified distinct sets of discriminatory proteins across plasma, tear fluid, and nasal secretions, indicating that each biofluid captured specific proteomic features associated with acute exercise. These biofluid-specific discriminatory patterns suggest that different sampling compartments provide complementary molecular information for characterizing exercise-associated proteomic alterations. Rather than representing validated exercise-responsive biomarkers, these proteins should be considered candidate molecular features that may contribute to the identification of exercise-associated proteomic signatures. Together with the complementary information obtained from multiple biofluids, these findings highlight the value of a multi-biofluid approach for gaining a more comprehensive understanding of exercise-related proteomic changes.

The present study has several methodological strengths. By integrating plasma with easily accessible mucosal biofluids, including tear fluid and nasal secretions, this study extends current exercise proteomic research beyond single-fluid analyses. This multi-biofluid design provides complementary molecular information and enables a broader characterization of acute exercise-associated proteomic alterations across biological compartments. In addition, the paired pre- and post-exercise sampling design within the same individuals minimized the influence of inter-individual variability and allowed a more direct assessment of exercise-associated proteomic changes. Despite these methodological strengths, several limitations of the present study warrant consideration. First, the findings are based on a paired within-subject pre-post design in five young, healthy females; therefore, the generalizability of the results to male populations and older adults remains to be established. The limited sample size inherent to this exploratory discovery study may have constrained statistical power; accordingly, differentially abundant proteins were identified using nominal *P*-values, and downstream functional analyses should be interpreted with appropriate caution. Second, potential pre-analytical influences related to biofluid sampling cannot be completely excluded despite the application of standardized collection procedures. Specifically, Schirmer strip-based tear collection may introduce a certain degree of reflex tear contribution due to ocular surface stimulation, while nasal lavage samples may be affected by variations in dilution factors related to endogenous secretion volume and mucosal conditions. Third, acute exercise-induced plasma volume alterations were not directly assessed in the present study. Although total protein normalization and MS intensity normalization were applied to reduce the influence of global abundance variation, potential effects of hemoconcentration on circulating protein profiles cannot be completely excluded. Finally, as a discovery-based DIA proteomic study, independent validation of the identified protein alterations was not performed; future studies involving larger independent cohorts and targeted validation approaches are required to further confirm their biological relevance.

## 5 Conclusion

In summary, this study provides a multi-biofluid proteomic characterization of acute exercise-associated molecular alterations by integrating paired plasma, tear fluid, and nasal secretion samples. Highly abundant proteins shared across the three biofluids were mainly associated with immune-related functions and extracellular protein regulation. Joint mixed-effects analysis further identified both common and biofluid-dependent exercise-associated responses among shared proteins, highlighting the distinct molecular characteristics captured by different biological compartments. Overall, this study demonstrates that acute exercise-associated proteomic changes can be characterized across multiple biological fluids, providing a broader molecular perspective on exercise responses.

## Data Availability

The datasets presented in this study can be found in online repositories. The names of the repository/repositories and accession number(s) can be found below: http://www.proteomexchange.org/, PXD074866.

## Supplementary material

Supplementary Figure 1 GO and KEGG enrichment analyses of biofluid-specific proteins across plasma, tear fluid, and nasal secretions. The figure is organized into three columns corresponding to plasma (left column), tear fluid (middle column), and nasal secretions (right column). **(A–C)** GO enrichment analysis of proteins uniquely detected in each biofluid. **(D–F)** KEGG pathway enrichment analysis of proteins uniquely detected in each biofluid.

Supplementary Figure 2 KEGG pathway enrichment analysis of upregulated proteins in nasal secretions.
