# Supplementary material for: Exploratory multi-biofluid proteomic profiling of acute exercise responses in plasma, tear fluid, and nasal secretions
Source: Front Physiol. 2026 Sep 23;17:1864411. doi: 10.3389/fphys.2026.1864411 (PMC13642130; doi:10.3389/fphys.2026.1864411)
Supplement: Supplementary file 1 [file DataSheet1.pdf]

Borg 6–20 Rating of Perceived Exertion (RPE) Record Form

Participant ID: 1 Date: 2025-5-23

Exercise protocol: acute aerobic exercise

Borg 6–20 RPE Scale

Score	Perceived exertion	Selection
6	No exertion at all	☐
7	Extremely light	☐
8		☐
9	Very light	☐
10		☐
11	Light	☐
12		☑
13	Somewhat hard	☐
14		☐
15	Hard	☐
16		☐
17	Very hard	☐
18		☐
19	Extremely hard	☐
20	Maximal exertion	☐

Borg 6–20 Rating of Perceived Exertion (RPE) Record Form

Participant ID: 2 Date: 2025-5-23

Exercise protocol: acute aerobic exercise

Borg 6–20 RPE Scale

Score	Perceived exertion	Selection
6	No exertion at all	☐
7	Extremely light	☐
8		☐
9	Very light	☐
10		☐
11	Light	☐
12		☐
13	Somewhat hard	☑
14		☐
15	Hard	☐
16		☐
17	Very hard	☐
18		☐
19	Extremely hard	☐
20	Maximal exertion	☐

Borg 6–20 Rating of Perceived Exertion (RPE) Record Form

Participant ID: 3 Date: 2025-5-23

Exercise protocol: acute aerobic exercise

Borg 6–20 RPE Scale

Score	Perceived exertion	Selection
6	No exertion at all	☐
7	Extremely light	☐
8		☐
9	Very light	☐
10		☐
11	Light	☐
12		☐
13	Somewhat hard	☑
14		☐
15	Hard	☐
16		☐
17	Very hard	☐
18		☐
19	Extremely hard	☐
20	Maximal exertion	☐

Borg 6–20 Rating of Perceived Exertion (RPE) Record Form

Participant ID: 4 Date: 2025-5-23

Exercise protocol: acute aerobic exercise

Borg 6–20 RPE Scale

Score	Perceived exertion	Selection
6	No exertion at all	☐
7	Extremely light	☐
8		☐
9	Very light	☐
10		☐
11	Light	☐
12		☑
13	Somewhat hard	☐
14		☐
15	Hard	☐
16		☐
17	Very hard	☐
18		☐
19	Extremely hard	☐
20	Maximal exertion	☐

Borg 6–20 Rating of Perceived Exertion (RPE) Record Form

Participant ID: 5 Date: 2025-5-23

Exercise protocol: acute aerobic exercise

Borg 6–20 RPE Scale

Score	Perceived exertion	Selection
6	No exertion at all	☐
7	Extremely light	☐
8		☐
9	Very light	☐
10		☐
11	Light	☐
12		☑
13	Somewhat hard	☐
14		☐
15	Hard	☐
16		☐
17	Very hard	☐
18		☐
19	Extremely hard	☐
20	Maximal exertion	☐
